# The EGF Receptor and HER2 Participate in TNF-****α****-Dependent MAPK Activation and IL-8 Secretion in Intestinal Epithelial Cells

**DOI:** 10.1155/2012/207398

**Published:** 2012-09-05

**Authors:** Humberto B. Jijon, Andre Buret, Christina L. Hirota, Morley D. Hollenberg, Paul L. Beck

**Affiliations:** Gastrointestinal Research Group, Health Sciences Centre, University of Calgary, Calgary, AB, Canada T2N 1N4

## Abstract

TNF-*α* activates multiple mitogen-activated protein kinase (MAPK) cascades in intestinal epithelial cells (IECs) leading to the secretion of interleukin 8 (IL-8), a neutrophil chemoattractant and an angiogenic factor with tumor promoting properties. As the epidermal growth factor receptor (EGFR) is a known transducer of proliferative signals and a potent activator of MAPKs, we hypothesized that the EGFR participates in TNF-dependent MAPK activation and IL-8 secretion by intestinal epithelial cells (IECs). We show that the EGFR is tyrosine-phosphorylated following treatment of IECs (HT-29 and IEC-6) with TNF-*α*. This requires EGFR autophosphorylation as it was blocked by the EGFR kinase inhibitor AG1478. Autophosphorylation was also inhibited by both a Src-kinase inhibitor and the metalloproteinase inhibitor batimastat. TNF treatment of IECs resulted in the accumulation of soluble TGF-*α*; treatment of IECs with batimastat suppressed TGF-*α* release and immunoneutralization of TGF-*α* resulted in decreased EGFR and ERK phosphorylations. TNF-*α* treatment of IECs resulted in an association between EGFR and HER2 and inhibition of HER2 using a specific inhibitor AG879 in combination with AG1478-suppressed TNF-*α*-dependent ERK phosphorylation and IL-8 release. Downregulation of HER2 via siRNA resulted in a significant decrease in ERK phosphorylation and a 50% reduction in IL-8 secretion.

## 1. Introduction

Inflammatory bowel diseases (IBDs), comprised of ulcerative colitis and Crohn's disease, are chronic, relapsing-remitting inflammatory diseases of unknown etiology. Current understanding suggests a critical role for the innate immune system in the context of a permissive genetic background and the intestinal microflora [1]. Interestingly, like other chronic inflammatory disorders, IBD is associated with an increased risk of cancer. In patients with ulcerative colitis particularly, the presence of either extensive or prolonged colonic disease can lead to a 20–30-fold increased risk of developing colorectal cancer (CRC) [2, 3]. 

 The mechanism(s) by which chronic inflammation contributes to carcinogenesis are poorly defined. Tumors, including CRC, are infiltrated by activated immune cells including T cells, neutrophils, macrophages, and dendritic cells which secrete various cytokines, chemokines, proteases, and growth factors. This results in the modification of the surrounding stroma creating an environment conducive to tumor growth, invasion, and eventual metastasis [2, 4–6].

 Tumor necrosis factor-alpha (TNF-*α*) is a proinflammatory cytokine known to play a central role in the development of intestinal inflammation and IBD [7]. Elevated serum levels of TNF-*α* have been demonstrated in IBD patients [8, 9], and anti-TNF therapies can be effective in the treatment of patients with otherwise refractory CD and UC [10–12]. Interestingly, TNF-*α* was recently shown to play a critical role in the development of colorectal cancer in an animal model of chemically induced colitis-associated cancer [2, 3, 13]. 

 TNF-*α* affects the growth, migration, differentiation, and function of intestinal epithelial cells (IECs) [14–18]. However, how TNF-*α* mediates these functional changes in IECs remains poorly understood. TNF-*α* is known to act through two distinct cell-surface receptors, a 55-KDa receptor and a 75-KDa receptor referred to as TNFR-I and TNFR-II, respectively, although most biological activities are attributed to the type I receptor [19, 20]. Historically, the first defined molecular target of TNF-*α* receptor signaling was the EGF receptor (EGFR) [21], a 170 kDa protein prototypical of a family of growth factor receptors characterized by a conserved N-terminal extracellular ligand-binding domain, a single transmembrane domain, and an intracellular C-terminus which possesses tyrosine kinase activity [22]. However, until recently the significance of TNF-dependant EGFR phosphorylation has remained obscure. The EGF receptor is a well-characterized transmitter of proliferation and differentiation signals, and a potent activator of the ERK MAPK pathway. Engagement of the EGF receptor results in its dimerization and activation of its intrinsic tyrosine kinase activity leading to receptor autophosphorylation on tyrosine residues [22, 23]. These phosphotyrosine residues then serve as docking sites for molecules containing specific domains involved in protein-protein interactions (e.g., Src-homology-2 (SH2) domains). Thus tyrosine phosphorylation of the EGFR is necessary for the recruitment and subsequent activation of multiple signaling pathways including the ERK pathway [22, 23]. 

In contrast to EGFRs, TNF-*α* receptors (TNFRs) do not possess any known catalytic activity and instead rely exclusively on adaptor molecules for the recruitment and transmission of extracellular signals [24]. Work over the last two decades has unveiled a unique set of intracellular signaling cascades downstream of TNF receptors, which elicit TNF-*α*-dependent cellular changes in a cell- and tissue-specific manner. TNF is a potent activator of MAPK signaling; however, the mechanisms whereby TNF-*α* activates the ERK MAPK pathway, remain poorly understood [19, 20]. GRB2, an adaptor molecule which couples receptor tyrosine kinase receptors to the MAPK pathway has been reported to associate with the type I TNF-*α* receptor, suggesting a direct link between TNFR-I and ERK [25]. In addition, RIP2 and MADD, two TNFR-I-interacting proteins, have been proposed to activate the ERK pathway in response to TNF-*α* [26, 27]. Also, the kinase and adaptor molecule KSR has recently been suggested to couple TNF receptors to ERK signaling in intestinal epithelial cells, leading to protection from cytokine induced apoptosis [28, 29]. Other groups have reported evidence for TNF-*α*-dependent EGFR transactivation and suggest that this event is required for ERK pathway activation in both hepatocytes and mammary epithelial cells [30, 31].

We have previously described the activation of the ERK signaling pathway in response to TNF-*α* in the transformed intestinal epithelial cell line HT-29 leading to expression of the angiogenic and chemotactic cytokine interleukin 8 (IL-8) [32]. EGFR gene amplification and overexpression are deemed important mechanisms leading to colonic epithelial transformation while IL-8 is believed to not only stimulate new blood vessel growth but also participates in the epithelial-mesenchymal transition in the colon [5, 33]. Therefore, EGFR transactivation leading to IL-8 secretion may not only contribute to inflammatory cell recruitment and activation in the context of IBDs but could also constitute an important component of colonic epithelial transformation. In this study we examined whether the EGF receptor is required for TNF-*α*-mediated activation of the ERK pathway leading to the secretion of IL-8 in intestinal epithelial cells. We report that maximal ERK activation and IL-8 secretion in response to TNF-*α* requires the release of TGF-*α* and the activation of the EGFR family of receptors.

## 2. Materials and Methods

### 2.1. Materials

 Unless otherwise stated all chemicals were purchased from Sigma (St. Louis, MO). 

### 2.2. Cell Culture

 HT-29 and IEC-6 cells were obtained from American Type Culture Collection (Rockwell, MA). HT-29 cells were cultured in RPMI 1640 media (Gibco, Burlington, Ontario) supplemented with 10% heat-inactivated fetal calf serum (Cansera, Rexdale, Ontario), 2 mmol/L glutamine, 1 mmol/L sodium pyruvate, 2% sodium bicarbonate, and 10 mmol/L HEPES. IEC-6 cells were cultured in DMEM supplemented with 5% fetal calf serum, 2 mmol/L glutamine, and 5 *μ*g/mL insulin. For experimental treatments, cells were grown in either 6 or 12 well tissue culture plates (Falcon, NJ).

 Confluent monolayers (passage 25–45) were incubated with human recombinant TNF-*α* (10 ng/mL, R&D systems, Minneapolis, MN) or epidermal growth factor (EGF, 50 ng/mL) in the presence or absence of the ERK pathway inhibitor PD98059 (Calbiochem, San Diego, CA), the platelet-derived growth factor (PDGF) receptor tyrosine kinase inhibitor AG1298, Src kinase inhibitor PP2 (Calbiochem, San Diego, CA), the tyrosine kinase inhibitor genistein (Calbiochem, San Diego, CA) the matrix metalloproteinase inhibitor batimastat (BB94) (Tocris, Ellisville, MO), the TNF-alpha converting enzyme (TACE) inhibitor TAPI-1 (Calbiochem, San Diego, CA), the EGF receptor tyrosine kinase inhibitor AG1478 (Calbiochem, San Diego, CA), and the HER2 receptor tyrosine kinase inhibitor AG879 or TGF-*α* neutralizing serum (R&D Systems, Minneapolis, MN). Cells were treated with the inhibitors for 30 mins prior to treatment with TNF-*α* or EGF. Control monolayers were treated with an equal volume of vehicle (DMSO for all inhibitors, PBS pH 7.4 for EGF and TNF-*α*). Prior to experiments designed to measure ERK activation or EGFR/HER2 transactivation, cells were incubated in serum-free media (OptiMEM, Invitrogen, Carlsbad, CA) overnight in order to reduce growth factor-mediated activation. All experiments were conducted in serum-free media.

### 2.3. Determination of IL-8 and TGF-*α* in Supernatants 

 For the purpose of measuring IL-8, HT-29 monolayers were stimulated with 10 ng/mL TNF-*α* or 50 ng/mL EGF for 3 hrs. IL-8 protein in supernatants was measured via ELISA as follows: 96 well Maxisorp ELISA plates (Nunclon, Rochester, NY) were coated with 4 *μ*g/mL capture monoclonal anti-IL-8 antibody (R&D Systems, Minneapolis, MN) in PBS (pH 7.4) overnight. Plates were then blocked overnight (5% sucrose, 0.05% sodium azide, 1% BSA in PBS pH 7.4). Plates were washed 4 times between all steps with 0.05% Tween-20 PBS pH 7.4. 100 *μ*L samples and standards (0–4000 pg/mL human recombinant IL-8, R&D Systems, Minneapolis, MN) were incubated in the plates overnight. Biotinylated polyclonal anti-IL-8 antibody (R&D Systems, Minneapolis, MN) was added (20 ng/mL in PBS pH 7.4) and plates incubated for 2 hrs. 100 *μ*L Streptavidin-HRP (Southern Biotechnology Associates, Birmingham, AL) was added for 1 hr, followed by development with 100 *μ*L TMBS (Calbiochem, San Diego, CA). Reaction was stopped with acid (0.5 M H_2_SO_4_) and plates read immediately at 450 nm using an ELISA plate reader (UV max, Molecular Devices, Sunnyvale, CA). All steps were carried out at room temperature. ELISA was sensitive to <30 pg/mL. TGF-*α* in cell culture supernatants was measured using a commercial TGF-*α* ELISA following manufacturer's instructions (R&D Systems, Minneapolis, MN).

### 2.4. Immunoprecipitation and Neutralization Studies

 Cells were grown in six well plates (100 mm dishes for IEC-6 cells) and treated in duplicate as described in figure legends (results). Cells were harvested in 200 *μ*L/well (500 uL/dish for IEC-6 cells) ice-cold modified RIPA buffer (250 mM NaCl, 50 mM HEPES, 0.5% NP40, 10% glycerol, 2 mM EDTA pH 8.0, 1 mM sodium orthovanadate, 1 mM PMSF, 10 *μ*g/mL leupeptin, 10 *μ*g/mL aprotinin) and sonicated on ice for 30 secs. Lysates were centrifuged at 4000 RPM for 2 min and supernatants transferred to new tubes. Protein concentrations were determined using a commercial Lowry Assay, (Biorad DC, Biorad, Hercules, CA). Protein concentrations were adjusted to the same concentration (5 mg/mL) then, 5 *μ*g of anti-EGFR, anti-her-2 antibody added (1 *μ*g/*μ*L, Santa Cruz Biotech, Santa Cruz, CA), or antiphosphotyrosine (4G10 monoclonal, kind gift from Dr. Stephen Robbins) and incubated on a rotator overnight at 4°C. Antibody was precipitated by the addition of 50 *μ*L of a 50% protein A/G-sepharose bead suspension for 2 hr at 4°C. Beads were washed 4 times with ice-cold modified RIPA buffer, supernatant was aspirated, then 60 *μ*L 2X protein sample buffer was added per sample. Samples were boiled for 5 min, centrifuged at 10000 rpm for 1 min, and proteins separated by SDS-PAGE as described above. For TGF-*α* immunoneutralization studies, HT-29 cells were treated with 1–10 *μ*g/mL anti-TGF-*α* or Ig control sera (R&D Systems, Minneapolis, MN) for 30 min prior to stimulation with TNF-*α* for 15 min. Cells were then harvested and analyzed for phospho-ERK content as described in the following.

### 2.5. Western Blotting

 Monolayers were stimulated with 10 ng/mL TNF-*α* or 50 ng/mL EGF and harvested in Mono Q buffer (1.08 g *β*-glycerophosphate, 38.04 mg EGTA, 0.5 mL Triton X-100, 200 *μ*L MgCl_2_ per 100 mL) at different times. Following sonication for 30 secs, samples were centrifuged at 12000 rpm for 1 min to remove insoluble material and protein concentrations were determined using a commercial Lowry Assay (Biorad DC, Hercules, CA) using BSA standards made in Mono Q buffer. Lysate concentrations were adjusted to ensure even protein loading, mixed with an equal volume of 2X protein sample buffer (130 mM Tris pH 6.8, 20% glycerol, 4% SDS, 5%  *β*-mercaptoethanol, trace bromophenol blue, 4 mM Sodium orthovanadate (Calbiochem, San Diego, MN), 2 *μ*M microcystin (Calbiochem, San Diego, MN)), boiled for 2 mins, and separated via electrophoresis (10% acrylamide gels). Proteins were transferred for 1.5 hrs (2 hrs for EGFR/HER2 immunoprecipitation experiments) at 400 mA in transfer buffer (25 mM Tris-base, 150 mM glycine, 10% methanol) onto a PVDF membrane (Millipore, MA). Membranes were blocked for 1 hr using 3% skim milk (5% BSA for antphosphotyrosine blots) and incubated overnight in primary antibody. The antibodies used were as follows: anti-ERK-1 (1 : 3000, rabbit, Upstate Biotech, Lake Placid, NY), anti-phospho-ERK 1/2 (1 : 1000, rabbit, New England Biolabs, Beverly, MA), anti-phosphotyrosine (1 : 1000, 4G10 monoclonal, kind gift from Dr. Stephen Robbins), anti-EGFR, and anti-HER2 (1 : 1000, Santa Cruz Biotech, Santa Cruz, CA). Secondary staining was conducted using HRP-conjugated goat sera specific for mouse or rabbit Ig as required (1 : 3000, Amersham, Baie d'Urfe, Quebec) followed by chemiluminescent detection using a commercial reagent following manufacturer's instructions (Lumilight, Roche, Laval, Quebec). Comparisons were made only among samples isolated and transferred together onto the same membrane. Multiple exposures were done to ensure that film was not overexposed. In order to confirm equal loading of protein, all western blots using phospho-specific antibodies were stripped and reprobed with antibody against the nonphosphorylated kinase. 

### 2.6. TACE Activity

HT-29 cells were incubated in serum-free media overnight, washed once with serum-free media, and stimulated with 10 ng/mL TNF-*α* for 15 mins. Cells were washed 2X with ice-cold PBS and harvested on ice. TACE activity was measured using a commercially available fluorimetric TACE assay kit (Sensolyte 520, AnaSpec, San Jose, CA) as per manufacturer's instructions. Fluorescence was measured every 5 mins for 3 hrs and plotted over time. Data represents fluorescence following 1 hr incubation with fluorescent substrate which is within the linear portion for all curves. 

### 2.7. HER2 siRNA Knockdown

Single-cell suspensions of HT-29 cells were prepared by trypsinizing 100 mm confluent monolayers. 5 × 10^5^ cells were transfected with 80 pmols siRNA reagent (control siRNA-A and HER2, Santa Cruz Biotech, Santa Cruz, CA) using Lipofectamine 2000 (Invitrogen, Carlsbad, CA) following manufacturer's instructions. Cells were cultured for a further 48 hrs in serum-free media prior to treatment with TNF-*α* as described in results and figure legends. 

### 2.8. Statistical Analysis

Unless otherwise stated, data shown in figures are representative experiments. Comparable results were obtained in additional experiments. Bar graphs are expressed as mean ± SD from at least three separate experiments. Differences between mean values were analyzed using the Student's *t*-test. *P* < 0.05 was considered statistically significant.

## 3. Results

### 3.1. EGF Rapidly Stimulates the ERK Pathway in HT-29 Cells

**Figure 1:**
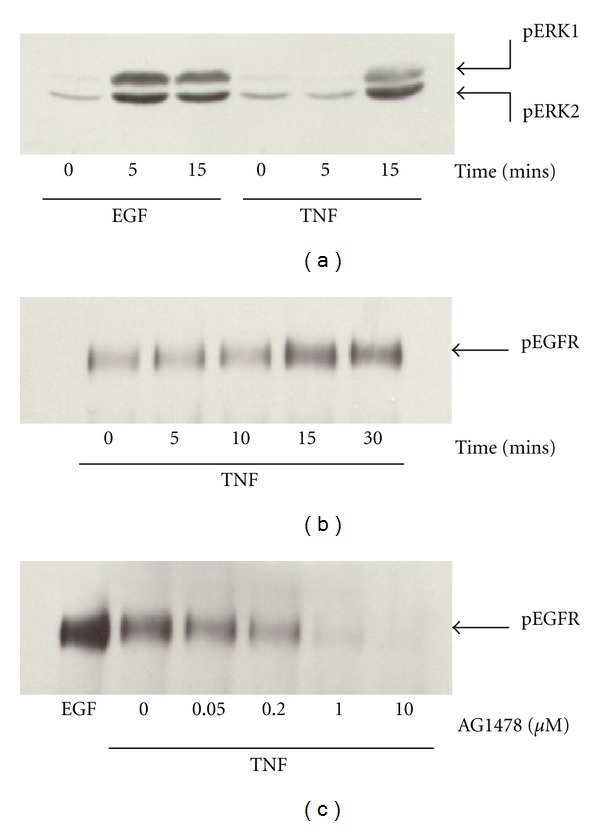
EGF rapidly stimulates the ERK pathway in HT-29 cells. HT-29 cells were cultured in serum-free media overnight and stimulated with 50 ng/mL EGF or 10 ng/mL TNF (a). ERK activation in response to TNF is relatively delayed (apparent by 15 mins) relative to EGF (apparent by 5 mins). (b) shows an antiphosphotyrosine blot of immunoprecipitated EGFR following stimulation of serum-starved HT-29 cells with TNF-*α*. TNF-*α* treatment results in the time-dependent tyrosine phosphorylation of the EGF receptor. (c) shows the effect of EGF receptor tyrosine kinase inhibition using the EGFR tyrosine kinase inhibitor AG1478. Cells were treated for 15 mins with AG1478 (0–10 *μ*M) and stimulated with 10 ng/mL TNF-*α* for 15 mins. AG1478 dose-dependently inhibits EGFR phosphorylation on tyrosine. Data are representative of at least three separate experiments.

 We have previously shown that TNF-*α* rapidly stimulates the phosphorylation (activation) of multiple MAPK pathways in HT-29 cells, including the ERK pathway leading to IL-8 secretion [32]. Previous studies have suggested an interaction between the EGFR and TNF-*α* signaling, some studies suggesting that the EGFR acts downstream of TNF receptors [15, 21, 34–38]. In that the EGFR is a potent activator of the ERK pathway in IECs, we sought to determine whether the EGFR couples TNF to ERK/MAPK signaling leading to IL-8 secretion [14, 15]. As shown in Figure 1(a), the kinetics of EGF-dependent ERK activation in HT-29 cells are consistent with the possibility that the EGFR couples TNF to ERK activation. ERK was rapidly activated following EGF treatment with significant ERK phosphorylation evident by 5 mins after stimulation whereas TNF-dependant ERK activation was only evident by 15 mins. 

### 3.2. TNF-*α* Stimulates EGFR Tyrosine Phosphorylation in HT-29 Cells

 Previous studies have described changes in EGFR tyrosine phosphorylation in response to TNF-*α* stimulation in various cell types [15, 21, 34–38]. Kaiser and Polk have previously reported a reduction in EGF-dependent EGFR tyrosine phosphorylation in response to TNF-*α* in intestinal epithelial cells [15, 16]. Argast et al. and Chen et al. on the other hand have recently reported EGFR transactivation in response to TNF-*α* in hepatocytes and mammary epithelial cells, respectively [30, 31]. They propose a similar model to that recently described for GPCR-mediated transactivation of growth factor receptors. This involves the extracellular release of growth factors via what is referred to as the “triple membrane passing signal” model of EGFR transactivation. Under this model, GPCR activation results in the activation of a membrane-bound matrix metalloproteinase (MMP) which then cleaves membrane-tethered EGFR ligands resulting in autocrine EGFR activation and Ras/ERK signaling [39–41]. We sought to examine whether a similar mechanism mediates ERK activation by TNF-*α* in intestinal epithelial cells. HT-29 cells were cultured in serum-free media overnight, stimulated with 10 ng/mL TNF-*α* for various times, and the EGF receptor immunoprecipitated. EGFR tyrosine phosphorylation was then assessed by western blotting using antiphospho-tyrosine sera. As shown in Figure 1(b), there was a low level of constitutive EGFR tyrosine phosphorylation in control cells which increased significantly following 15 mins treatment with TNF-*α*.

**Figure 2:**
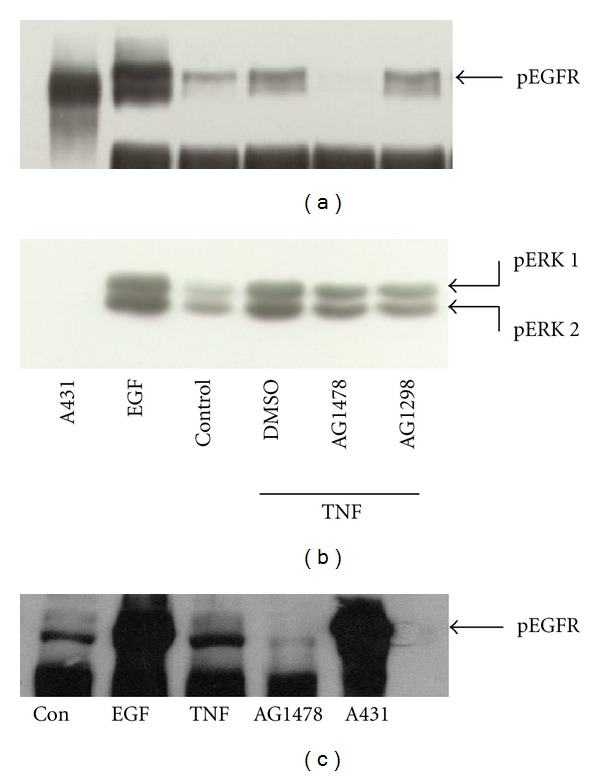
EGFR tyrosine phosphorylation is inhibited by the EGF receptor tyrosine kinase inhibitor AG1478 (10 *μ*M) but not the PDGF receptor inhibitor AG1298 (5 *μ*M) (a). In contrast, only a modest decrease in Erk1/2 phosphorylation was noted in response to pretreatment of HT-29 cells with either of these agents (b). Serum-starved cells were pretreated for 30 mins prior to stimulation with TNF-*α* for 15 mins, and EGFR tyrosine phosphorylation and ERK phosphorylation were assessed as described in *Materials and Methods*. Transactivation of the EGFR in response to TNF-*α* was also observed in the rat intestinal epithelial cell line IEC-6 (c). IEC-6 cells were treated with 10 ng/mL TNF-*α* for 15 mins in the presence or absence of 1 *μ*M AG1478. Data are representative of at least three experiments.

To determine whether the increase in tyrosine phosphorylation of the EGFR observed following TNF-*α* treatment requires the intrinsic kinase activity of the EGFR (transactivation), HT-29 cells were treated as above, except cells were incubated with the EGF receptor tyrosine kinase inhibitor AG1478 for 15 mins prior to TNF-*α* stimulation. As shown in Figure 1(c), EGFR phosphotyrosine content was dose-dependently reduced in the presence of AG1478. This effect was evident at 50 nM AG1478 with complete reduction apparent between 1 and 10 *μ*M AG1478. AG1278 (5 *μ*M), a PDGF-receptor tyrosine kinase inhibitor which is structurally similar to AG1478, did not affect EGF receptor tyrosine phosphorylation (Figure 2(a)). Interestingly, despite almost complete inhibition of EGFR phosphorylation, AG1478 had a modest effect on ERK phosphorylation (Figure 2(b)). TNF-*α*-dependant EGFR transactivation was also observed in the rat intestinal cell line IEC-6 (Figure 2(c)) suggesting that TNF-dependent EGFR transactivation is conserved across intestinal epithelial cell lines. On the other hand, there is a lack of correlation between the effects of AG1478 on EGFR phosphorylation and ERK activation. 

### 3.3. TNF-Dependent EGFR Transactivation Is Matrix Metalloproteinase Dependent

**Figure 3:**
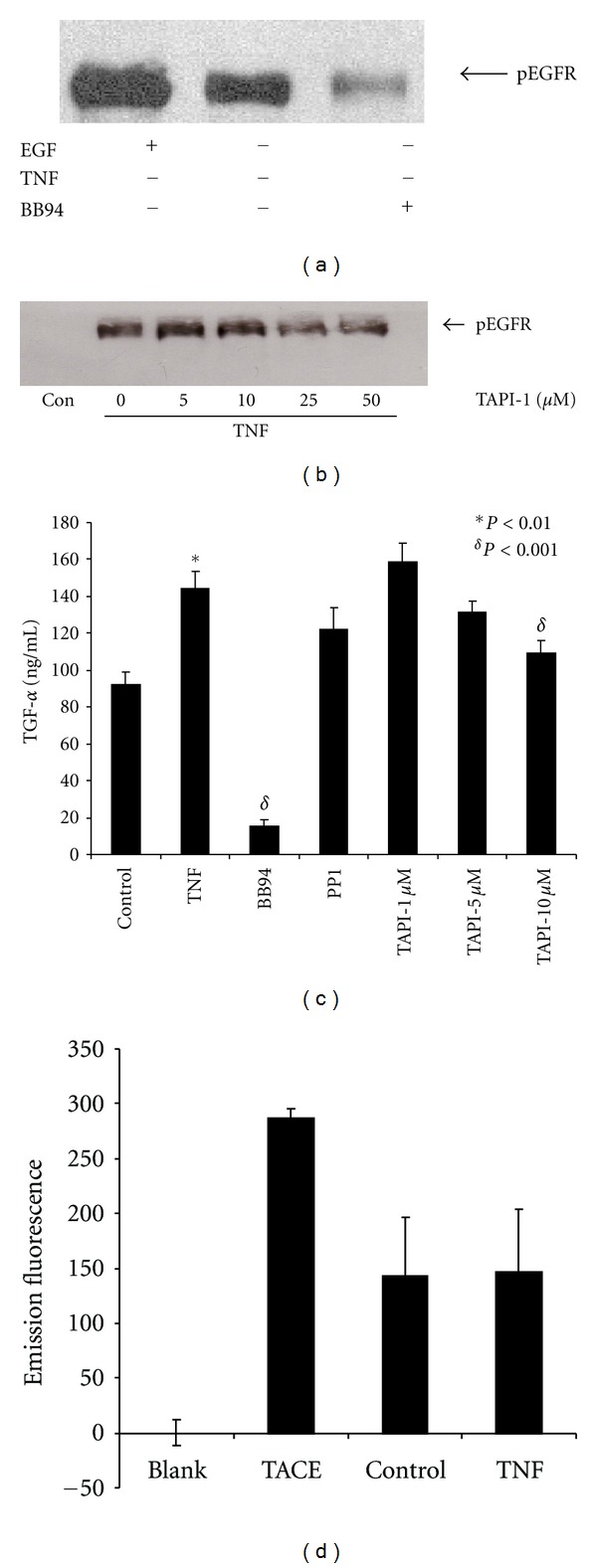
TNF-dependent EGFR transactivation requires metalloproteinase activity and results in TGF-*α* release. Serum-starved cells were treated for 30 min with the metalloproteinase inhibitor BB94 (batimastat, 10 *μ*M) (a), or increasing concentrations of the TNF-converting enzyme (TACE) inhibitor TAPI-1 (b), and stimulated with 10 ng/mL TNF-*α* for 15 mins. EGFR tyrosine phosphorylation was assessed as described in Section 2. EGFR tyrosine phosphorylation is significantly reduced in the presence of BB94 and to a lesser extent by TAPI-1. (c) shows the effect of BB94 and TAPI-1 pretreatments on TNF-stimulated TGF-*α* release. Serum-starved cells were pretreated for 30 mins with BB94 or TAPI-1, stimulated with TNF-*α* for 3 hrs, and TGF-*α* measured via ELISA. (d) shows total TACE activity as measured using either recombinant TACE or membrane preparations from vehicle and TNF-treated HT-29 cells using a fluorescent substrate. Cells were pretreated with vehicle or TAPI-1 for 30 mins prior to stimulation with TNF-*α* (10 ng/mL) for 15 mins (see Section 2). Data are representative of at least three experiments.

 We next examined whether MMP activity is required for EGFR transactivation in response to TNF-*α* in HT-29 cells. Cells were serum-starved overnight and treated for 15 mins with 10 ng/mL TNF-*α* in the presence or absence of the pan-MMP inhibitor batimastat (BB94, 10 *μ*M). As shown in Figure 3(a), treatment with BB94 resulted in almost complete inhibition of EGFR tyrosine phosphorylation in response to TNF-*α*, suggesting that EGFR tyrosine kinase activation in response to TNF-*α* requires MMP activity.

 We next sought to identify the MMP responsible for TNF-dependent EGFR transactivation. TNF-*α*-converting enzyme (TACE) is a metalloproteinase which derives its name from its ability to cleave membrane-bound TNF-*α* leading to TNF-*α* release, but it also cleaves multiple EGFR ligands including amphiregulin, HB-EGF, epiregulin, and TGF-*α* [42]. TACE is expressed in HT-29 cells where it participates in TNF-*α*-stimulated TNF-*α* release [43]. We therefore examined whether TACE is required for TNF-dependent EGFR transactivation. As shown in figure 3B, pretreatment of HT-29 cells with the TACE-specific inhibitor TAPI-1 attenuated EGFR phosphorylation following TNF-*α* treatment. 

 TGF-*α* has previously been implicated in TNF-*α*-stimulated EGFR transactivation [31, 44]. We therefore stimulated HT-29 cells with TNF-*α* and measured TGF-*α* in the culture media. As shown in Figure 3(c), treatment with TNF-*α* resulted in a 60% increase in soluble TGF-*α* compared to unstimulated controls. Pretreatment of cells with BB94 completely blocked TNF-*α*-stimulated TGF-*α* release as well as basal TGF-*α* release in unstimulated cells. On the other hand, pretreatment of HT-29 cells with increasing concentrations of the TACE inhibitor TAPI-1 had a dose-dependant effect on TNF-stimulated TGF-*α* release but did not alter basal TGF-*α* production (Figure 3(c)). We next measured TACE activity in control and TNF-stimulated cells using a fluorescent peptide substrate harbouring a TACE cleavage site. Interestingly, TACE activity did not change in response to TNF-*α* treatment (Figure 3(d)).

### 3.4. Tyrosine Kinase Inhibitors Inhibit EGFR Transactivation in Response to TNF-*α*

**Figure 4:**
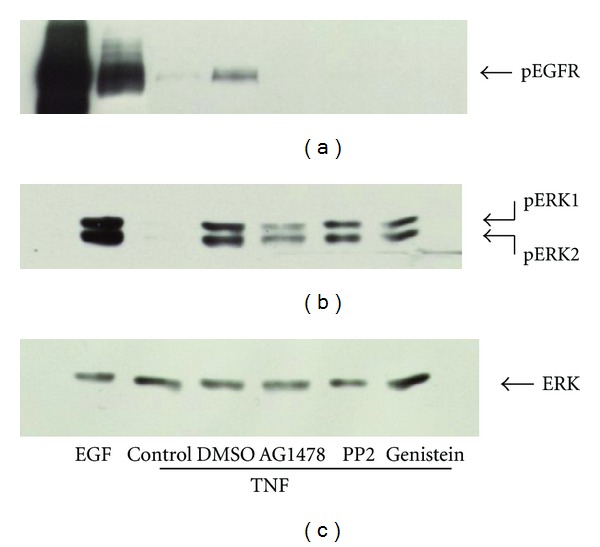
Tyrosine kinase inhibitors inhibit EGFR phosphorylation in response to TNF-*α*. HT-29 cells were treated with 2 *μ*M PP2 (Src-kinase inhibitor), 100 *μ*M genistein (tyrosine kinase inhibitor), or 10 *μ*M AG1478 for 15 mins prior to 15 mins of TNF-*α* treatment. The EGF receptor was immunoprecipitated and tyrosine phosphorylation assessed (a). Both PP2 and genistein abrogated TNF-*α*-dependent EGFR tyrosine phosphorylation. In contrast, neither PP2 nor genistein had an appreciable effect upon TNF-stimulated ERK1/2 phosphorylation (b) and (c). Data are representative of at least three experiments.

 The sensitivity of TNF-*α*-dependent EGFR phosphorylation to batimastat suggests that, similar to GPCRs, TNF-*α* utilizes a “triple membrane passing signal” mechanism in order to activate the EGFR. Unlike GPCRs, however, TNF-*α* does not trigger changes in intracellular calcium in HT-29 cells (data not shown); thus it is unlikely that TNF would act via Pyk, a calcium-dependent kinase suggested to play a role in other systems such as that of carbachol-stimulated EGFR transactivation in T84 intestinal epithelial cells [39]. Instead we asked whether tyrosine kinases such as Src family kinases are involved as has been suggested in other cell types [45]. HT-29 cells were treated with 10 *μ*M AG1478, 2 *μ*M PP2 (a Src-kinase inhibitor), or 100 *μ*M genistein (tyrosine kinase inhibitor) for 15 mins prior to treatment with TNF-*α* for 15 mins. The results of this experiment are shown in Figure 4(a). As before, TNF-*α* treatment resulted in increased EGFR tyrosine phosphorylation and this was blocked by AG1478. Interestingly, PP2 also abrogated EGFR tyrosine phosphorylation as well as the phosphorylation on tyrosine residues on proteins that coprecipitate with the EGFR (data not shown). This was also true of genistein, a broad specificity tyrosine kinase inhibitor, although genistein had a smaller effect upon the phosphotyrosine content of coprecipitating proteins. These results suggest the participation of Src-like kinases in relaying the signal that links TNF-*α* to the EGFR. In parallel experiments, we looked at the effects of these inhibitors upon TNF-stimulated ERK phosphorylation (Figure 4(b)). Similarly, PP2 and genistein had almost no effect upon ERK activation despite having completely abrogated EGFR phosphorylation (Figure 4(b)).

### 3.5. Neutralization of TGF-*α* Blocks Both EGFR Transactivation and ERK Signaling

**Figure 5:**
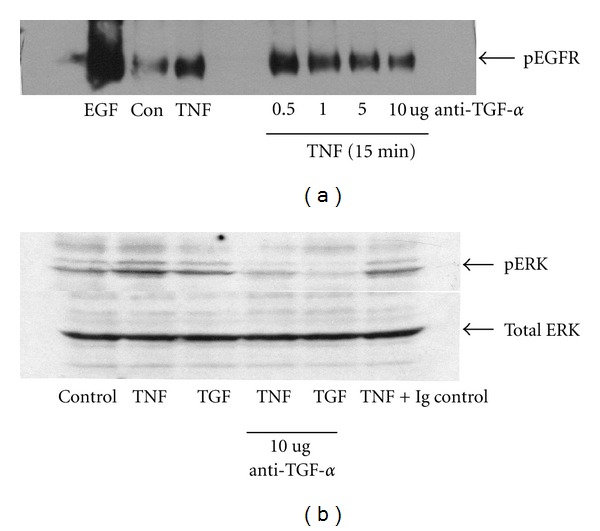
TGF-*α* release is required for TNF-*α*-stimulated EGFR transactivation and Erk1/2 phosphorylation. Serum-starved HT-29 cells were pretreated with increasing concentrations of TGF-*α*-neutralizing serum or Ig control and stimulated with TNF-*α* for 15 mins. EGFR was immunoprecipitated and phosphotyrosine content determined by western blotting (a). (b) shows the effect of TGF-*α*-neutralizing serum on TNF-stimulated Erk1/2 phosphorylation. Data are representative of at least 3 separate experiments.

 Having observed increased TGF-*α* release in response to TNF-*α* and considering the ability of a metalloproteinase inhibitor to attenuate both TGF-*α* release and EGFR phosphorylation, we next asked whether specific blockade of TGF-*α* using a neutralizing antibody could block both EGFR and ERK activations. HT-29 cells were incubated with increasing concentrations of TGF-*α* neutralizing sera or isotype control and both EGFR tyrosine phosphorylation and ERK activation examined. As shown in Figure 5(a), anti-TGF-*α* dose-dependently blocked EGFR tyrosine phosphorylation. This was paralleled by a significant reduction in ERK phosphorylation (Figure 5(b)).

### 3.6. HER2 Associates with EGFR and Participates in TNF-*α*-Dependent ERK Activation

 EGFR is a member of the structurally related ErbB family of transmembrane receptor tyrosine kinases, which also includes HER2 (Neu/ErbB2), HER3 (ErbB3), and HER4 (ErbB4) [46]. Heterodimerization between ErbB family members is common and adds to the diversity of signals which can be elicited by multiple ligands with different binding affinities. HER2 is an orphan receptor and frequently partners with other ErbB family members. Zhou and Brattain demonstrated synergy between EGFR and HER2 tyrosine kinase inhibitors towards the induction of apoptosis in human colon cancer cell lines [47]. In this study, EGFR transactivation and ERK activation could both be blocked by neutralizing TGF-*α*, in contrast to AG1478 which does not block ERK activation to the same degree as it blocks EGFR phosphorylation, suggesting that TGF-*α* may activate another EGFR family receptor leading to the activation of ERK. We therefore asked whether HER2, in association with EGFR, participates in TNF-*α* stimulated ERK activation.

**Figure 6:**
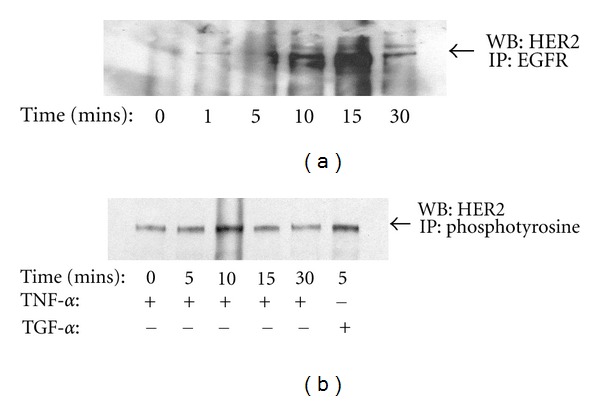
TNF treatment stimulates EGFR-HER2 heterodimerization, and HER2 tyrosine phosphorylation. HT-29 cells were serum-starved for 24 hrs prior to stimulation with 10 ng/mL TNF-*α*. EGFR was immunoprecipitated, and coprecipitating HER2 was measured via western blotting (a). HT-29 cells were serum-starved for 24 hrs, stimulated with 10 ng/mL TNF-*α* for 15 mins, and phosphotyrosine-containing proteins immunoprecipitated. Samples were separated by SDS-PAGE and HER2 content quantified by western blotting (b). Figures are representative of at least 3 separate experiments.

 To answer this question, we first stimulated HT-29 cells with TNF-*α* for various times and immunoprecipitated the EGFR. These immunoprecipitates were then probed for the presence of HER2. As shown in Figure 6(a), TNF-*α* treatment resulted in the time-dependant recruitment of HER2 to EGFR with peak association at 15 mins. Interestingly, this association is transient as it is no longer evident by 30 mins. Next, we assessed whether HER2 becomes phosphorylated on tyrosine residues in response to TNF-*α*. For this purpose serum-starved HT-29 cells were stimulated as before and tyrosine-phosphorylated proteins immunoprecipitated using antiphosphotyrosine sera. Samples were then probed via western blotting using anti-HER2 sera. As shown in Figure 6(b), HER2 phosphotyrosine content was significantly increased 10 mins after stimulation with TNF-*α* and after 5 mins of stimulation with TGF-*α*.

**Figure 7:**
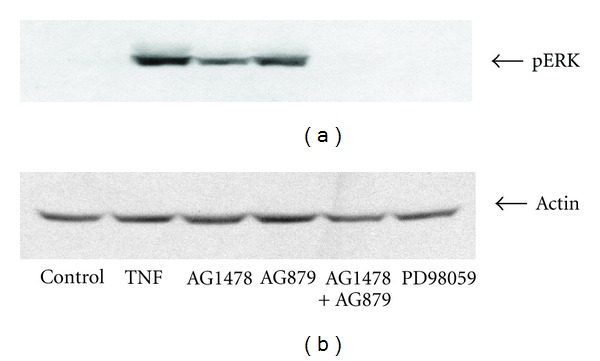
Inhibition of HER2 tyrosine kinase activity (2.5 *μ*M AG879) in addition to that of EGFR results in greater inhibition of ERK1/2 phosphorylation as compared to EGFR inhibition alone (10 *μ*M) (a) in HT-29 cells. (b) is a loading control, 25 *μ*M PD98058. Figure is representative of three separate experiments.

 We next asked whether inhibition of HER2 in combination with inhibition of the EGFR would result in greater inhibition of ERK activation than inhibition of the EGFR alone. HT-29 cells were incubated in the presence or absence of 5 *μ*M AG1478 and 2.5 *μ*M of the HER2-specific inhibitor AG879 prior to stimulation with TNF-*α*. As shown in Figure 7, combined inhibition of HER2 and the EGFR resulted in greater inhibition of ERK signaling as compared to EGFR inhibition alone.

### 3.7. EGF Receptor and HER2 Tyrosine Kinase Inhibitors Block TNF-*α*-Stimulated IL-8 Secretion by HT-29 Cells

**Figure 8:**
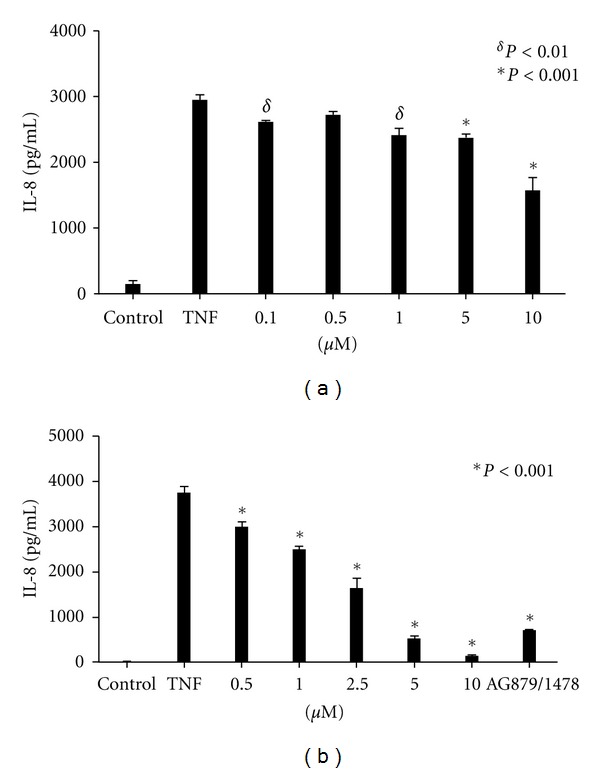
Effect of EGFR and HER2 inhibition on TNF-*α*-stimulated IL-8 secretion. HT-29 cells were treated with increasing doses of AG1478 for 30 mins prior to 6 hr stimulation with 10 ng/mL TNF-*α* (a). HT-29 cells were treated with increasing doses of the HER2 inhibitor AG879 or a combination of AG1478 and AG879 (b). Secreted IL-8 was measured via ELISA. Results are representative of three separate experiments. *P < 0.01, ^*δ*^ *P* < 0.001.

 In a previous study we showed a requirement for ERK in TNF-*α*-stimulated IL-8 secretion by intestinal epithelial cells [32]. We therefore asked whether inhibition of EGFR tyrosine kinase activity would decrease TNF-stimulated IL-8 secretion. HT-29 cells were treated with increasing doses of AG1478 for 15 mins followed by treatment with 10 ng/mL TNF-*α* for 6 hrs. The amount of secreted IL-8 was then measured in the supernatants via ELISA. As shown in Figure 8(a), TNF-*α*-stimulated IL-8 release was inhibited only at 10 *μ*M AG1478 (~50%, *P* < 0.001). On the other hand, while 1 *μ*M AG1478 was sufficient to completely block EGFR phosphorylation (Figure 1(c)), it had no effect on IL-8 secretion. We next tested the effect of HER2 inhibition upon IL-8 secretion. As shown in Figure 8(b), the HER2 inhibitor AG879 dose-dependently inhibited TNF-induced IL-8 secretion. Further, combined AG879 and AG1478 at submaximal doses inhibited IL-8 secretion in an additive manner.

### 3.8. HER2 siRNA Blocks TNF-*α*-Stimulated ERK Activation and IL-8 Secretion in HT-29 Cells

**Figure 9:**
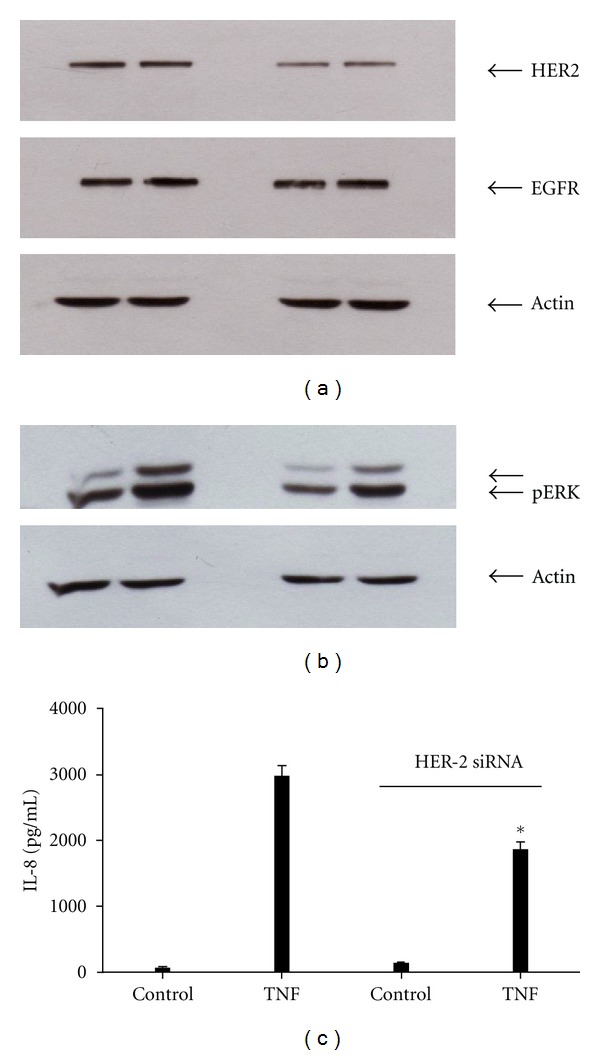
HER2 knockdown using siRNA attenuates TNF-dependant ERK activation and significantly inhibits IL-8 secretion. HT-29 cells were transfected with control or HER2-specific siRNA and incubated in serum-free media for 48 hrs. Cell lysates were prepared for western blotting as described in Section 2. HER2-specific siRNA reduced HER-2 protein expression but did not alter EGFR expression (a). siRNA-transfected cells were then stimulated with 10 ng/mL TNF-*α* for 15 mins, or 6 hrs and ERK phosphorylation, and IL-8 secretion measured as described previously ((b) and (c) resp.). Figures are representative of at least 3 separate experiments. *P < 0.001.

Tyrosine kinase inhibitors selective for EGFR and HER2 suggested a role for these receptors in TNF-stimulated ERK activation and IL-8 secretion. To further demonstrate a role for ErbB2/Her2 in this process we made use of siRNA specific to HER2. HT-29 cells were transfected with HER2-specific siRNA for 48 hrs and both EGFR and HER2 protein levels determined by immunoblotting (Figure 9). As shown in Figure 9(a), HER2 protein expression levels were significantly decreased by treatment with HER2 siRNA. In contrast, the expression of EGFR was unaffected by treatment with HER2-specific siRNA (Figure 9(a) middle). We next took HER2 siRNA-treated HT-29 cells, stimulated them with TNF-*α* for 15 mins, and determined the levels of phospho-ERK. As shown in Figure 9(b), downregulation of HER2 via siRNA significantly reduced ERK activation in response to TNF-*α*. Lastly, HT-29 cells were transfected with HER2 siRNA for 48 hrs, stimulated for an additional 12 hrs with TNF-alpha, and IL-8 protein secretion measured via ELISA. As shown in Figure 9(c), inhibition of HER2 protein expression via siRNA led to a profound reduction in IL-8 secretion in response to TNF-*α* treatment.

## 4. Discussion

Various studies have described the phosphorylation of the EGF receptor in response to TNF-*α*. This has been shown to occur on tyrosine residues, threonine residues, or both and to result in different outcomes depending on the cell type studied. Donato et al. examined multiple fibroblast cell lines and suggested that phosphorylation of the EGF receptor occurs predominantly on threonine residues and results in a reduction in EGF receptor affinity in cell lines susceptible to TNF-*α*-mediated cytotoxicity [34]. On the other hand, Guazzoni et al. reported inhibition of EGFR tyrosine phosphorylation which was accompanied by a decrease in EGF receptor tyrosine kinase activity in a fibroblast cell line [35]. Further, Murthy et al. reported EGFR tyrosine phosphorylation in response to IL-1 and TNF-*α* in the intestinal epithelial cell line Caco-2, an event which mimics the effects of the EGFR ligand EGF [37]. In this last study, Murthy and coworkers identified 2 peaks in EGFR tyrosine phosphorylation in response to TNF, one at 30 mins and the other at 6.5 hrs. Interestingly, it was determined that the early peak was ligand independent whereas the later peak could be abolished using a receptor blocking antibody [37]. 

In this study we provide evidence that TNF activates one or more metalloproteinases leading to the release of TGF-*α* in intestinal epithelial cells. TNF-dependant EGFR phosphorylation was abrogated by the pan-MMP inhibitor BB94 (Figure 3(a)) and BB94 profoundly reduced TGF-*α* release both basally and in response to TNF-*α* (Figure 3(c)). Blocking TGF-*α* in turn led to reduced EGFR activation and ERK phosphorylation (Figures 5(a) and 5(b)). In a previous study, we demonstrated that ERK activation was necessary for maximal IL-8 secretion through a mechanism involving the stabilization of IL-8 mRNA. Thus, TNF activates multiple signaling cascades including the I*κ*K/NF*κ*B, p38 and ERK pathways which act at different points to stimulate maximal IL-8 release: stimulating NF*κ*B nuclear translocation [48, 49], increasing NF-*κ*B transcriptional activity [48, 49] and stabilizing IL-8 mRNA message [32]. 

 Previously, Janes et al. showed that TNF-*α* stimulates EGFR transactivation and the ERK signaling pathway in HT-29 cells via an autocrine loop involving TGF-*α*. In this study they showed that blocking TGF-*α*/EGFR signaling enhanced TNF-*α*/IFN-*γ*-induced apoptosis. They used an EGFR-neutralizing antibody (C225, Cetuximab/Erbitux) to completely block TNF-stimulated EGFR phosphorylation and downstream signaling. Our data with AG1478, the EGFR inhibitor, was initially very difficult to interpret. We observed a complete blockade of EGFR phosphorylation with AG1478; however, we could at best only partially block TNF-dependant ERK activation and had almost no effect upon IL-8 secretion with this drug alone. In the study by Janes et al, they pretreated cells with IFN-*γ* before all their experiments in order to enhance apoptosis in response to TNF-*α*. IFN-*γ* pretreatment is a key difference between their experimental design and ours; however, we were unable to completely block ERK activation or IL-8 secretion with AG1478 with or without IFN-*γ* pretreatment (unpublished data). However, using combined EGFR and HER2 inhibition, we can achieve greater ERK and IL-8 inhibition than either inhibitor alone. Interestingly, inhibition of HER2 using AG879 alone had a profound effect upon IL-8 secretion (~50% reduction at 2.5 *μ*M), but combined inhibition using both AG1478 (1 *μ*M) and AG879 (2.5 *μ*M) resulted in greater than 80% inhibition. This may represent a nonspecific effect on the part of our inhibitors or a greater role for the EGFR/HER2 receptor complex upon IL-8 secretion, which may involve the activation of pathways other than the MEK/ERK pathway. Recently, Sethi et al. have suggested that the EGFR can stimulate NF*κ*B activation independent of IKK through the phosphorylation of I*κ*B on tyrosine 42 [50]. Although this pathway may contribute to NF*κ*B activation and IL-8 secretion in IECs, there is significant evidence pointing to the importance of IKK-dependent I*κ*B phosphorylation and degradation leading to NF*κ*B activation and proinflammatory gene expression in these cells [49]. Future experiments will look at the effect of EGFR/HER2 inhibition upon NF*κ*B activation and I*κ*B degradation.

While this paper was in preparation, Hobbs and coworkers have shown TNF transactivation of the EGFR stimulates COX-2 expression in mouse intestinal cells. They provide evidence to suggest the participation of Src and p38, kinases in an MMP-independent manner. In our hands, a Src inhibitor (PP2) and a tyrosine kinase inhibitor (genistein) completely blocked EGFR phosphorylation and yet had no effect upon ERK activation (Figure 4). It would be expected that if Src or a related kinase lies upstream of MMP(s) and TGF-*α* release, Src inhibition should result in reduced EGFR and Her-2 activities leading to decreased ERK activation. Perhaps in the absence of Src, TNF-stimulates ERK activity through a yet undetermined mechanism. Alternatively, there could exist two parallel pathways leading to EGFR transactivation downstream from TNF-*α*, one Src-dependent, and one MMP-dependent. TNF-*α* has been shown to activate both in other systems [30, 31, 37, 44]. Janes and coworkers and now us have shown a requirement for TGF-*α* in TNF-dependent ERK activation using human HT-29 cells; perhaps there exist cell line/species-specific differences that underlie the noted discrepancies between these studies 

 TACE seemed like a good candidate to be the MMP activated by TNF leading to TGF-*α* release. The TACE inhibitor TAPI-1 inhibits TNF-stimulated TGF-*α* release but this is most evident at high concentrations. In addition, TNF failed to stimulate TACE activity. Interestingly, there is precedent for this as Myhre et al. have recently shown that TACE may be regulated by at the level of cellular localization as opposed to enzymatic activity [51].

 In the present study we focused on the role of the EGFR/HER2 signaling pathway in TNF-stimulated IL-8 secretion. However, this pathway is likely to contribute to many aspects of TNF signaling in IECs. Both the EGFR and TNF are known to profoundly affect intestinal epithelial cell function. Of particular interest in this regard, Janes et al. have shown that this pathway may modulate IEC apoptosis which may have implications towards the development of cancer in the context of inflammation [44]. Work by Yamaoka et al. has suggested that TNF-dependant transactivation of the EGFR/Her2 heterodimer activates Akt thus activating an antiapoptotic program which protects IECs from TNF-dependant apoptosis [52]. Likewise, in our study we provide evidence that the EGFR may contribute towards the production of the potent angiogenic chemokine IL-8. IL-8 not only acts as a potent neutrophil chemoattractant but also has been shown to be the most bioactive chemoattractant for microvascular endothelial cells in the context of human IBD, contributing to the development of an abnormal mucosal vascular bed in the context of intestinal inflammation [53]. Importantly, polymorphisms within the loci coding for IL-8 receptors A and B have recently been identified in genomewide association studies supporting an important role for IL-8 in the pathogenesis of IBD [54]. 

 IL-8 and other cytokines such as IL-6 have been shown to play a critical role in tumor growth in multiple cancer models independent of inflammation such as in Ras-driven models of cancer [6]. Il-8 has been shown to recruit regulatory T cells which via their immunosuppressive abilities may contribute to tumor escape from immune surveillance [55]. Interestingly, therapies targeting both EGFR and Her 2 have been shown to normalize tumor vascularization [56]. Thus, IL-8 secretion in the context of inflammation may act to stimulate angiogenesis in the absence of mutant Ras and therapies targeting EGFR signaling may act in part by blocking IL-8 production.

The fact that EGFR/HER2 participates in TNF signaling may have several important therapeutic implications. First, it suggests that therapies which target the EGFR/HER2 may potentially affect immune responses in the gut. Second, EGFR/HER2 activation by TNF may contribute to inflammation induced carcinogenesis. This possibility will have to await testing *in vivo* to see the effect of EGFR/Her2 signaling inhibition in the context of a colitis-induced cancer model. Third, attempts at abrogating EGFR signaling in the context of TNF-*α* signaling must keep in mind the participation of other EGFR binding partners such as HER2.
